# Cardiac dysfunction due to mitochondrial impairment assessed by human iPS cells caused by *DNM1L* mutations

**DOI:** 10.1038/s41390-025-04045-6

**Published:** 2025-04-23

**Authors:** Madori T. Osawa, Yasunori Fujita, Kazuki Kagami, Masataka Ito, Yoshiteru Tamura, Shoichiro Tateishi, Junya Take, Fumi Hirose, Hidetoshi Hagiwara, Kohsuke Imai, Daisuke Yoshinaga, Shiro Baba, Mitsujiro Osawa, Hiroko Harashima, Kei Murayama, Yuko Akioka, Akira Ohtake, Ikuro Suzuki, Takeshi Adachi, Takeru Yamazaki, Satoshi Arai, Shiro Matsumoto, Tetsuya Kitaguchi, Megumu K. Saito, Ikuroh Ohsawa, Shigeaki Nonoyama

**Affiliations:** 1 Department of Pediatrics, National Defense Medical College, Saitama, Japan; 2 Department of Pediatrics, Faculty of Medicine, Saitama Medical University, Saitama, Japan; 3 Biological Process of Aging, Tokyo Metropolitan Institute for Geriatrics and Gerontology, Tokyo, Japan; 4 Department of Cardiovascular Medicine, National Defense Medical College, Saitama, Japan; 5 Department of Developmental Anatomy and Regenerative Biology, National Defense Medical College, Saitama, Japan; 6 Department of Pediatrics, Graduate School of Medicine, Kyoto University, Kyoto, Japan; 7 Department of Clinical Application, Center for iPS Cell Research and Application, Kyoto University, Kyoto, Japan; 8 Department of Clinical Genomics, Faculty of Medicine, Saitama Medical University, Saitama, Japan; 9 Diagnostics and Therapeutics of Intractable Diseases, Intractable Disease Research Center, Juntendo University, Graduate School of Medicine, Tokyo, Japan; 10 Department of Metabolism, Chiba Children’s Hospital, Chiba, Japan; 11 Department of Electronics, Graduate School of Engineering, Tohoku Institute of Technology, Miyagi, Japan; 12 Nano Life Science Institute (WPI-NanoLSI), Kanazawa University, Kanazawa, Japan; 13 Department of Pediatrics, Faculty of Life Sciences, Kumamoto University, Kumamoto City, Kumamoto Japan; 14 Laboratory for Chemistry and Life Science, Institute of Innovative Research, Tokyo Institute of Technology, Kanagawa, Japan

## Abstract

**Background:**

*DNM1L* encodes dynamin-related protein 1, which plays an important role in mitochondrial and peroxisomal division. The *DNM1L* mutation leads to cardiac dysfunction in patients and animal models. However, the mechanism of cardiac dysfunction caused by *DNM1L* mutation has not been elucidated clearly at least in the studies of human cardiomyocytes.

**Methods:**

We established human induced pluripotent stem cells (hiPSCs) from two pediatric patients with *DNM1L* mutation. The hiPSCs were differentiated into hiPSC-derived cardiomyocytes (hiPS-CMs). Mitochondrial morphology and function, cardiomyocyte Ca^2+^ dynamics, and contractile and diastolic function of hiPS-CMs were analyzed.

**Results:**

The morphology of the mitochondria was abnormally elongated in patient-derived hiPS-CMs. The mitochondrial membrane potential and oxygen consumption rate were significantly decreased, resulting in reduced ATP production. In the analysis of Ca^2+^ dynamics, the 50% time to decay was significantly longer in patient-derived hiPS-CMs than in healthy control. High-precision live-imaging system analysis revealed that contractile and diastolic function was significantly impaired under isoproterenol stimulation.

**Conclusion:**

*DNM1L* mutations cause mitochondrial impairment with less production of ATP in cardiomyocytes. This leads to abnormal intracellular Ca^2+^ dynamics, resulting in contractile and diastolic dysfunction.

**Impact:**

- *DNM1L* mutations was identified in two pediatric patients who developed cardiac dysfunction and human induced pluripotent stem cells (hiPSCs) were established from these two patients and differentiated into hiPSC-derived cardiomyocytes (hiPS-CMs).
- *DNM1L* mutations induced abnormal mitochondrial morphology, mitochondrial dysfunction, and insufficient ATP production in hiPS-CMs. In addition, hiPS-CMs with *DNM1L* mutation showed abnormal Ca^2+^ kinetics and impaired contractile and diastolic function.
- This is the first study that elucidate the mechanism of cardiac dysfunction caused by *DNM1L* mutations by using hiPSCs.

## Introduction

Mitochondrial diseases are characterized by their clinical, biochemical, and genetic complexity. Most patients suffer from childhood with typical symptoms such as growth disorder, developmental delay, muscle weakness, and cardiomyopathy. Mitochondria are intracellular organelles and form highly dynamic networks through a balance of fusion and fission. *DNM1L* encodes the dynamin-related protein 1 (Drp1), a member of the dynamin superfamily of GTPases. Drp1 plays important roles in mitochondrial and peroxisomal division and mitochondrial trafficking and distribution.^1–3^ Drp1 accumulates on the mitochondrial outer membrane and interacts with receptors such as fission protein 1 (FIS1), mitochondrial fission factor, and mitochondrial dynamics proteins of 49 kDa and 51 kDa (MiD49 and MiD51, respectively).^4^ Drp1 forms homodimers and self-assembles to form a ring-shaped band around mitochondria. Contraction of this band causes mitochondrial fission.^2^ This process maintains mitochondrial quality control because senescent mitochondria are detached and processed by mitophagy. When this process is disrupted, elongation and abnormal distribution of mitochondria and peroxisomes occur.^2,3,5–7^

Drp1 is mainly composed of a GTPase, stalk (middle), variable, and GED domains.^8^ The GTPase domain is responsible for binding to receptors on the mitochondrial outer membrane and is involved in conformational changes that accelerate mitochondrial fission.^9^ On the other hand, the stalk domain is involved in Drp1 self-assembly and oligomerization,^10,11^ and the GED domain stimulates GTPase activity and contributes to the formation and stability of homodimeric complexes.^12^ The variable domain is a target site for SUMOylation and GlcNAcylation and is involved in regulating GTPase activity.^13^

*DNM1L* mutations have been reported to cause developmental delays, intractable epilepsy, and encephalopathy in children and neonates. Heterogeneous de novo missense variants have been identified in most patients^11,14–36^ and have been reported to cause mitochondrial elongation owing to a dominant-negative effect.^11,14,15,17,18,24,27^ A few cases of biallelic compound heterozygous and homozygous recessive inheritance have been described.^16,19,23^

In addition to neurological disorders, Ahrafian et al.^37^ reported that mice with a *DNM1L* mutation developed dilated cardiomyopathy and showed decreased ATP production in the myocardium. Kageyama et al.^38^ also showed that left ventricular contractility was reduced in Drp1-knockout mice. While neurological phenotypes are severe, cardiac function has not been evaluated precisely in the *DNM1L* mutated patients so far. Recently, some patients with the mutation were reported that they had cardiac dysfunction.^35,36^ However, the mechanism of cardiac dysfunction has not been elucidated at least in human cardiomyocytes with *DNM1L* mutations. Therefore, this study focuses on revealing the mechanisms of cardiac dysfunction in human cardiomyocytes. For this purpose, we established human induced pluripotent stem cells (hiPSCs) from pediatric patients with *DNM1L* mutation and differentiated them into hiPSC-derived cardiomyocytes (hiPS-CMs) in vitro.

## Materials and methods

### Ethical considerations

This study was approved by the Ethics Committee of the National Defense Medical College (approval number 4845) and was conducted in accordance with the principles of the Declaration of Helsinki. Since the patients involved in this study were children, the research contents were explained in writing and verbally to the parents, and a signed informed consent was obtained.

### Establishment of hiPSC

hiPSCs were generated from two unrelated individuals harboring *DNM1L* mutation. The hiPSC lines were established as previously reported.^39^ Briefly, we obtained fibroblasts from patients and reprogrammed them by transfecting Oct3/4, Sox2, Klf4, L-Myc, Lin28, mp53DD, and EBNA1 with episomal vectors into hiPSCs. Three colonies were created in patient 1 and two in patient 2. Of these, one colony from each patient was selected for stable proliferation cell line which differentiated into cardiomyocytes more effectively. hiPSCs established from peripheral blood mononuclear cells of healthy female Asians were purchased from Phenocell (Provence-Alpes-Côte d’Azur, France) and used as a control. The control iPSCs does not have *DNM1L* mutations assessed by Sanger sequencing (data not shown).

### hiPSC passage and culture

We seeded 1.5–2.0 × 10^5^ hiPSCs/well into a 6-well plate pre-coated with Matrigel (CORNING, Corning, NY) and maintained in mTeSR1^TM^ (Stem Cell Technologies, Vancouver, Canada) medium supplemented with 5 mM of the ROCK inhibitor Y-27632 (FUJIFILM WAKO, Osaka, Japan). On the following day, the medium was replaced with fresh mTeSR1 ^TM^ without Y-27632. The medium was changed daily, and at 70–80% confluence, the cells were passaged.

### Cardiomyocyte differentiation and purification

Cadiomyocyte differentiation was induced with glycogen synthase kinase-3 (GSK3) and Wnt inhibitors as previously reported^40^ with slight modification. Briefly, hiPSCs were dissociated with accutase (Innovative Cell technologies, San Diego, CA) and replated on wells on a 12-well plate pre-coated with Matrigel in 1.5 ml/well mTeSR1^TM^ medium supplemented with 5 μM of Y-27632 (FUJIFILM WAKO) at a density of 0.5–2.5 × 10^5^ cells per each well on 4 days before the initiation of cardiomyocyte differentiation (Day-4). On Day-3, Day-2, Day-1, the medium was replaced with fresh mTeSR1^TM^ without Y-27632. On Day 0, to initiate cardiomyocyte induction, the medium was replaced with 1.5 ml/well RPMI-1640 (Thermo Fisher Scientific, Waltham, MA) with B27 supplement (RPMI/B27) without insulin (Thermo Fisher Scientific), supplemented with 8 μM CHIR99021 (GSK3 inhibitor) (Selleckchem, Houston, TX). On day 1, the medium was replaced with fresh 1.5 ml/well RPMI/B27 without insulin. On Day 3, the medium was changed with 1.5 ml/well RPMI/B27 without insulin, supplemented with 5 μM IWP2 (Wnt inhibitor) (Tocris Bioscience, Bristol, UK). On Day 5, the medium was replaced with fresh 1.5 ml/well RPMI/B27 without insulin. On Day 7 and every 2 days thereafter, the medium was changed with 1.5 ml/well RPMI/B27 with insulin. By around day 14, the cells were beating spontaneously.

To purify hiPS-CMs, hiPS-CMs at 20–30 days of the differentiation were cultured in lactate medium, containing 4 mM lactate (FUJIFILM WAKO) in glucose-free DMEM (Sigma Aldrich, St. Louis, MO), for 7 days.^41^ The lactate medium was changed every 2–3 days to remove dead cells. We confirmed the purity of hiPS-CMs by the positivity of cardiac troponin T by fluorescence-activated cell sorting (FACS) analysis (see Supplementary Methods). Only hiPS-CMs that reached a purity of 80% or higher were used for further experiments. After purification, hiPS-CMs were cultured in the maintenance medium; DMEM-F12 (Sigma Aldrich) with 2% fetal bovine serum (Thermo Fisher Scientific) containing Glutamax^TM^ (Thermo Fisher Scientific), non-essential amino acids (Thermo Fisher Scientific), 2-mercaptoethanol (Sigma Aldrich), and penicillin/streptomycin (Thermo Fisher Scientific). All hiPS-CMs used in the experiments had been differentiated for more than 60 days.

### Mitochondrial staining of hiPS-CMs

Mitochondrial morphology was visualized using MitoTracker Red (MTR) (Thermo Fisher Scientific). The hiPS-CMs were seeded on 35-mm glass-bottom tissue culture plates (Iwaki, Shizuoka, Japan) in the maintenance medium without phenol red and incubated at 37 °C, 5% CO_2_ for 4–7 days. Subsequently, the cells were incubated with 200 nM MTR for 30 min at 37 °C, 5% CO_2_ in the dark. Nuclei were stained with NucBlue (Thermo Fisher Scientific). Live imaging of hiPS-CMs was performed using fluorescence confocal microscopy (TCS SP8X, Leica, Wetzlar Germany) at 40× magnification. Fluorescence images were obtained under the following conditions: frame size: 1024 × 1024, excitation: 578 nm (MTR) and 405 nm (NucBlue), emission filter set: 588–655 nm (MTR) and 423-485 nm (NucBlue), and laser power: 0.15% (MTR) and 0.2% (NucBlue).

### Transmission electron microscopy

The hiPS-CMs were fixed with 2% paraformaldehyde and 2.5% glutaraldehyde, post-fixed with 2% osmium tetroxide, and embedded in epoxy resin. Ultrathin sections were cut, stained with uranyl acetate, and observed under a transmission electron microscope (JEM-1400Plus, JEOL, Tokyo, Japan). Mitochondrial length was measured by scaling the longitudinal length of the mitochondria using the ImageJ software (NIH, Bethesda, MD).

### Transfection of patient-derived hiPS-CMs with mCherry-Drp1 plasmid

Patient-derived hiPS-CMs were seeded on triple-well glass-bottom tissue culture plates (Iwaki) in the maintenance medium and incubated at 37 °C, 5% CO_2_ for 4–7 days. Subsequently, cells were transfected with the complex of mCherry-Drp1 (mCh-Drp1) plasmid (plasmid #49152, Addgene, Watertown, MA) and Transporter^TM^ 5 Transfection Reagent (Polysciences, Warrington, PA) at 37 °C, 5% CO_2_ for 24-48 h. After washing with phosphate-buffered saline, cells were incubated with 200 nM MitoTracker Green (MTG) (Thermo Fisher Scientific) in a fresh medium for 30 min at 37 °C, 5% CO_2_ in the dark. Live imaging of transfected hiPS-CMs was performed using fluorescence confocal microscopy at 40× magnification with 3× zoom under the following conditions: frame size: 1024 × 1024, excitation: 490 nm (MTG) and 588 nm (mCh-Drp1), emission filter set: 505–555 nm (MTG) and 597-690 nm (mCh-Drp1), and laser power: 4.0% (MTG) and 2.0% (mCh-Drp1). Mitochondrial length was measured by scaling the longitudinal length of the mitochondria using Mitochondria Analyzer, a plugin for ImageJ software.

### Analysis of mitochondrial membrane potential

Mitochondrial membrane potential (ΔΨm) was estimated using JC-1 (Dojindo, Kumamoto, Japan). The hiPS-CMs were seeded on 35-mm glass-bottom tissue culture plates in the maintenance medium and incubated at 37 °C, 5% CO_2_ for 4–7 days. Subsequently, the cells were incubated with 4 μM JC-1 for 30 min at 37 °C, 5% CO_2_ in the dark. The hiPS-CMs were visualized using fluorescence confocal microscopy (TCS SP8X) at 40× objective. Fluorescence images were obtained under the following conditions: frame size: 1024 × 1024, excitation: 535 nm (red) and 488 nm (green), emission filter set: 560–610 nm (red) and 500–550 nm (green), and laser power: 0.03% (red) and 0.8% (green). The ratio of red/green fluorescence intensity of JC-1 was measured using the ImageJ software.

### Measurement of oxygen consumption rate

Oxygen consumption rate (OCR) was measured using a high-resolution respirometry (Oxygraph-2k, Oroboros, Innsbruck, Austria). The hiPS-CMs were detached from the 24-well plates using 1 mg/dL collagenase B (Roche, Roswell, GA) and Accumax (Innovative Cell Technologies, San Diego, CA). The number of cells was counted using an automatic cell counter (Thermo Fisher Scientific). The cells were washed twice with maintenance medium and suspended with MiR05 mitochondrial respiration medium (3 mM MgCl_2_, 60 mM K^+^-lactobionate, 20 mM taurine, 10 mM KH_2_PO_4_, 20 mM HEPES, 110 mM D-sucrose, 0.5 mM EGTA, pH adjusted to 7.1 with KOH at room temperature, and 1 g/L bovine serum albumin, essentially free of fatty acids). We measured the real-time OCR of hiPS-CMs using the Oroboros Oxygraph SUIT protocol with slight modification.^42^ Cell membranes of hiPS-CMs were permeabilized with 5 μg/mL digitonin, and mitochondrial respiration was established by adding 5 mM pyruvate and 2 mM malate, followed by 2.5 mM ADP, to measure the oxidative phosphorylation (OXPHOS) capacity of complex I (OXPHOS complex I), driven by the NADH-related substrates. To measure maximal OXPHOS capacity (OXPHOS complex I + II), 10 mM succinate was added. The OCR was estimated as pmol/ (s × million cells). Subsequently, 0.5 μM rotenone was added to inhibit complex I and measure complex II-linked OXOPHOS capacity. Finally, 2.5 μM antimycin A was added to inhibit complex III and measure mitochondrial residual oxygen consumption.

### Measurement of ATP production

To transfect hiPS-CMs with the expression vector for mitochondrial targeting MaLionR (MitoMAR),^43^ the hiPS-CMs were seeded on 35-mm glass-bottom tissue culture plates in the maintenance medium and incubated at 37 °C, 5% CO_2_ for 4–7 days. Subsequently, cells were transfected with the complex of expression vector and polyethylenimine (Polysciences) at 37 °C and 5% CO_2_ for 6 h. After washing with phosphate-buffered saline, the cells were cultured in a fresh medium at 30 °C for 48 h. Red fluorescence derived from MitoMAR was observed at 37 °C under a confocal microscope (FV1200, Olympus, Tokyo, Japan) equipped with a 60× oil-immersed lens in the time-lapse mode. During the observation, the cells were stimulated with oligomycin at a final concentration of 25 μM. Fluorescence images were obtained under the following conditions: frame size: 512 × 512, excitation: 561 nm, emission filter set: 575–675 nm, laser power: 1.5%, time per picture: 1.64 s, interval: 10 s, and number of pictures: 250.

### Ca^2+^ imaging of hiPS-CMs

The hiPS-CMs were seeded to 22-mm slide-glass dishes, incubated in the maintenance medium at 37 °C, 5% CO_2_ for 4–7 days and loaded with 5 μM Fluo-4 AM (DOJINDO, Kumamoto, Japan) dissolved in the maintenance medium in the dark for 30 min. The maintenance medium contains 120 mM NaCl, 14.3 mM NaHCO_3_, 0.45 mM NaH_2_PO_4_, 0.5 mM Na_2_HPO_4_, 4.2 mM KCl, 1.2 mM CaCl_2_, 0.5 mM MgCl_2_, 0.4 mM MgSO_4_, and 15 mM HEPES (pH 7.4). The cells were stimulated using platinum field-stimulation electrodes at pacing intervals of 600 ms and 400 ms (beating rates of 100 and 150 bpm, respectively), and calcium fluorescent signals were recorded using a fluorescence microscope (Keyence BZ-X710, Osaka, Japan). F/F0, the ratio of the peak-to-base fluorescence intensity, and 50% time to decay (T50) were calculated.^44^

### Motion analysis of hiPS-CMs

The hiPS-CMs were seeded in a 24-well plate with iCell cardiomyocyte maintenance medium (FUJIFILM Cellular Dynamics, Tokyo, Japan) and incubated for 4–7 days. Contractile and diastolic function were quantified using a high-precision live imaging system (SI8000, SONY, Tokyo, Japan). The plate seeded with cardiomyocytes was placed in a shooting unit and allowed to stand for 30 min until the samples stabilized. Following stabilization, motion images were recorded for 10 s. Subsequently, isoproterenol was administered (0.03, 0.1, 0.3, and 1.0 μM). Each concentration of isoproterenol was applied for 5 min, and video images were recorded for 10 s. Phase-contrast video imaging was performed using a 4× objective at 150 frames/s and a resolution of 2048 × 2048 pixels. The beating of cardiomyocytes was captured and the contraction and relaxation speeds during the beat were plotted at steady state and in tachycardia state induced with isoproterenol. We obtained a series of hiPS-CMs parameters from the motion waveforms, as previously reported.^45^ The parameters were maximum contraction speed (MCS), which represented contractile properties; maximum relaxation speed (MRS), which represented relaxation properties; and beating rate.

### Statistical analysis

Data are presented as mean ± standard error of the mean (SEM). The normality of data distribution was evaluated using the Shapiro-Wilk test to determine whether parametric or nonparametric tests should be performed. Normally distributed were analyzed using one-way analysis of variance followed by Tukey’s multiple comparisons test. Meanwhile, non-normally distributed data were analyzed using the Kruskal–Wallis test followed by Dunn’s multiple comparison test. Mixed-effects analysis was used for repeated measures. All statistical analyses were performed using GraphPad Prism version 9.5.1 (GraphPad Software, San Diego, CA). Results were considered statistically significant at *p* < 0.05.

## Results

### Patient characteristics

The characteristics of each patient are summarized in Supplementary Table S1.^20,35^ Mutation sites and domains of two patients in this study and previous reports are shown in Supplementary Fig. S1. The clinical course of these two patients suggested the development of some kind of cardiomyopathy, and especially in patient 2, the mitochondrial disease was suspected due to metabolic abnormalities accompanied by hyperlactemia. We established hiPSCs from these patients and differentiated them into hiPS-CMs.

#### Patient 1

A 6-month-old boy was hospitalized due to hypotonia and infantile spasm. Cerebral atrophy and developmental regression progressed. Echocardiography revealed myocardial thickening, pericardial effusion, and a mild decrease in cardiac contractility with an ejection fraction of 56% at the onset of pneumonia. The patient died of aspiration pneumonia at the age of 1 year and 6 months. A pathological autopsy revealed fibrosis and degeneration of cardiomyocytes, indicating cardiomyopathy. Whole-exome sequencing (WES) analysis revealed a de novo missense mutation (c.1217T>C, p.Leu406Ser) in *DNM1L* (Supplementary Fig. S2a).

#### Patient 2

A 20-month-old, previously healthy boy presented with high-grade fever, was admitted to emergency center. Because respiratory and heart failure progressed rapidly, extracorporeal membrane oxygenation support was initiated. His ejection fraction was reduced to 40%, and he had metabolic acidosis with hyperlactemia, hypoglycemia, and hyperammonemia, which led to early suspicion of mitochondrial disease. WES revealed a de novo missense mutation (c.1757C>A, p.Thr586Lys) in *DNM1L* (Supplementary Fig. S2b).

### Patient-derived hiPS-CMs exhibit elongated mitochondrial morphology

**Fig. 1:**
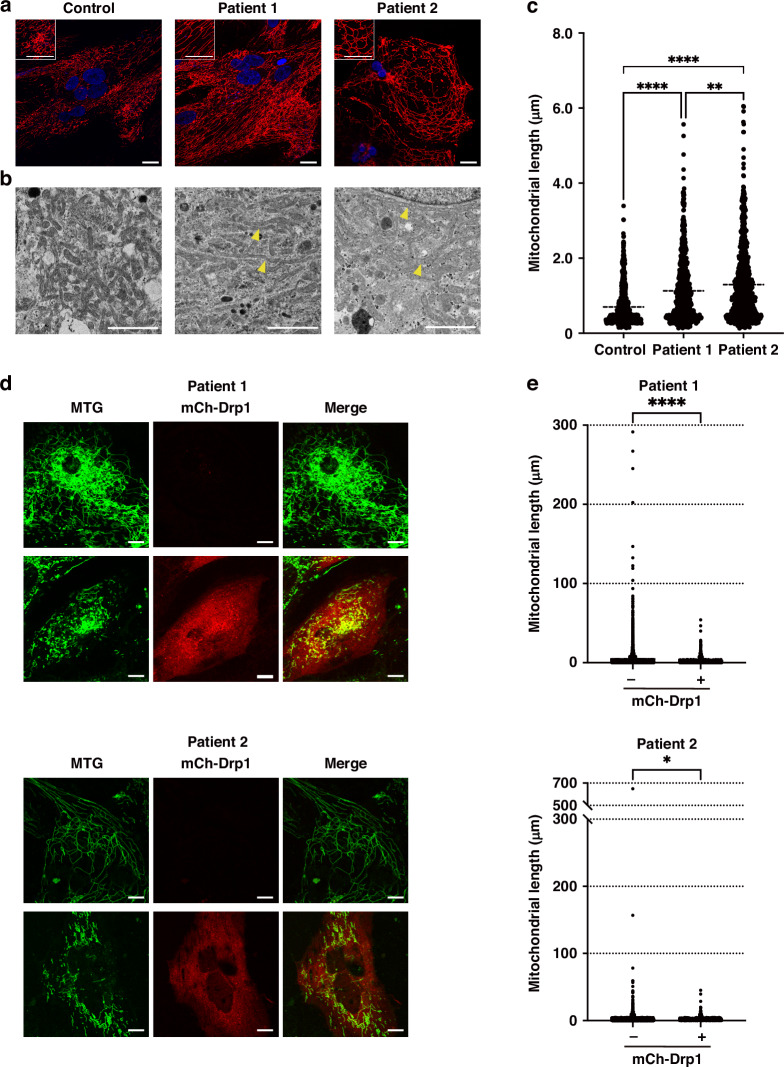
Comparison of mitochondrial morphology in hiPS-CMs. **a** Representative images taken by mitochondrial staining of hiPS-CMs with MTR. Scale bar: 25 μm. **b** TEM images of hiPS-CMs. Scale bar: 2 μm. Arrow heads indicate tubular, highly elongated mitochondria. **c** Mitochondrial length of hiPS-CMs in TEM images. The mitochondrial length was significantly elongated in patient-derive hiPS-CMs (control: 0.699 ± 0.019 μm [*n* = 722], patient 1: 1.127 ± 0.031 μm [*n* = 750], patient 2: 1.296 ± 0.036 μm [*n* = 788]). *n* = number of mitochondria in 5 cells. **p < 0.01, ****p < 0.0001. **d** Representative confocal microscope images of mCh-Drp1 expression and mitochondrial morphology in each patient-derived hiPS-CMs. The mitochondria were stained with MTG. Scale bar: 10 μm. **e** Mitochondrial morphology of mCh-Drp1-expressing hiPS-CM was significantly shorter and fragmented compared to non-expressing hiPS-CM (patient 1 mCh-Drp1(-): 4.598 ± 0.285 μm [*n* = 2527], mCh-Drp1(+): 2.002 ± 0.121 μm [*n* = 1146], patient 2 mCh-Drp1(-): 2.270 ± 0.226 μm [*n* = 3132], mCh-Drp1(+): 1.345 ± 0.062 μm [*n* = 1635]). *n* = number of mitochondria of 5 cells. *p < 0.05, ****p < 0.0001.

Mitochondrial stainings with MTR and electron micrographs showed a distinctly elongated morphology in patient-derived hiPS-CMs with the *DNM1L* mutation, unlike in control hiPS-CMs (Fig. 1a, b). Quantification of the mean longitudinal mitochondrial length in electron micrographs revealed that mitochondria in patient-derived hiPS-CMs were significantly longer than those in control (Fig. 1c). The elongated filamentous mitochondria were densely packed within the cell, implying an increased mitochondrial volume in patient-derived hiPS-CMs. To investigate whether this mitochondrial abnormality was due to the *DNM1L* mutation, we transfected each patient-derived hiPS-CM with the mCh-Drp1 plasmid to overexpress mCh-Drp1, which can compensate for the endogenous wild-type Drp1 defect.^46,47^ mCh-Drp1-expressing hiPS-CMs had significantly shorter mitochondrial lengths than those not expressing mCh-Crp1 (Fig. 1d, e). Although reduced expression of Drp1 inhibits mitochondrial fission, but Drp1 expression was unaffected in patient 1-derived hiPS-CM and rather increased in patient 2-derived hiPS-CM (Fig. S3a, b). Phosphorylation of Drp1 at serine 616 (S616) and serine 637 (S637) is involved in the initiation and inhibition of mitochondrial fission, respectively.^48,49^ However, phosphorylation at S616 was unaffected in patient-derived hiPS-CMs and phosphorylation at S637 was rather reduced (Fig. S3c, d).

### Patient-derived hiPS-CMs exhibit reduced ΔΨm and OCR

**Fig. 2:**
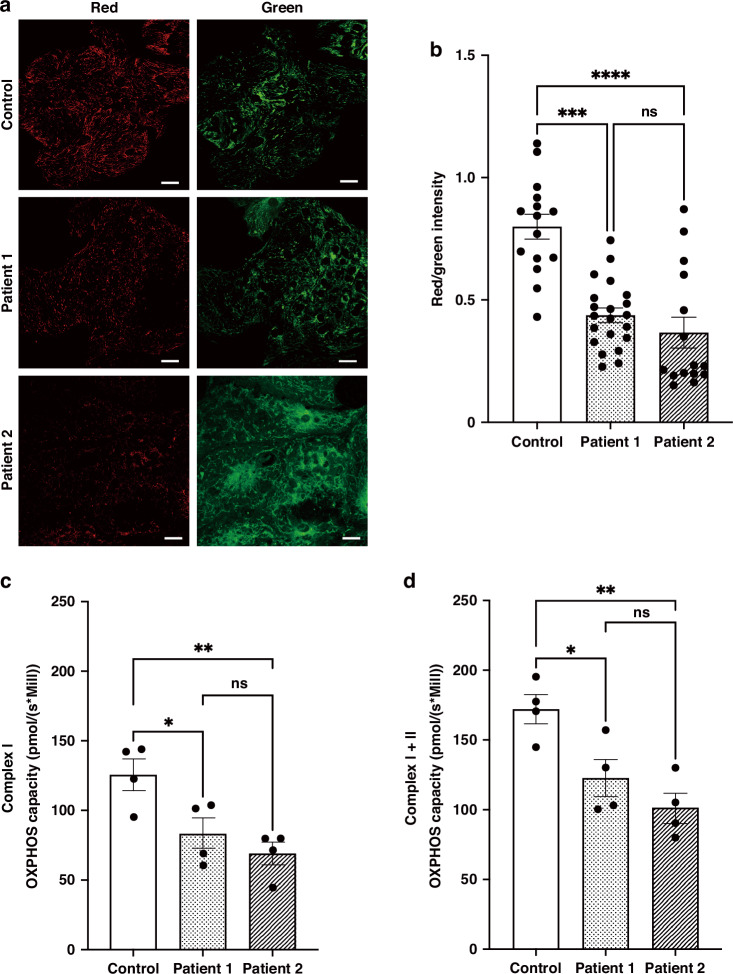
Evaluation of ΔΨm and OCR of patient-derived hiPS-CMs. **a** Representative fluorescence images of JC-1 stained hiPS-CMs. Aggregated JC-1 (red) and monomeric JC-1 (green) indicate polarized and depolarized mitochondria, respectively. Scale bar: 25 μm. **b** Ratio metric measurement (red/green fluorescence intensity) using JC-1. The ratio of red/green intensity is significantly lower in patient-derived hiPS-CMs than in control (control: 0.800 ± 0.051 [*n* = 15], patient 1: 0.438 ± 0.030 [*n* = 21], patient 2: 0.367 ± 0.063 [*n* = 15]). *n* = number of fields of view. ***p < 0.001, ****p < 0.0001, ns: no significant. **c**, **d** OCR of hiPS-CMs was measured using a high-resolution respirometry. OXPHOS of complex I was measured by adding 5 mM pyruvate and 2 mM malate, followed by 2.5 mM ADP (**c**). OXPHOS of complex I + II was measured by further addition of 10 mM succinate (**d**). The OCR is significantly reduced in patient-derived hiPS-CMs in OXPHOS complex I (control: 126.20 ± 11.33 [*n* = 4], patient 1: 83.83 ± 11.05 [*n* = 4], patient 2: 69.08 ± 8.36 [*n* = 4] pmol/[s × Mill]) and OXPHOS complex I + II (control: 172.10 ± 10.45 [*n* = 4], patient 1: 122.70 ± 13.29 [*n* = 4], patient 2: 101.40 ± 10.87 [*n* = 4] pmol/[s × Mill]), respectively. *n* = number of cell pellets. *p < 0.05, **p < 0.01, ns no significant.

To evaluate mitochondrial activity in hiPS-CMs, we monitored ΔΨm using JC-1, a cationic carbocyanine dye (green fluorescence), which exhibited potential-dependent accumulation in mitochondria and started forming J aggregates (red fluorescence). A higher ratio of red fluorescence to green fluorescence intensity (red/green intensity) indicated a higher ΔΨm. The ratio of red/green intensity of the patient-derived hiPS-CMs was significantly lower than that of control hiPS-CMs (Fig. 2a, b), indicating that patient-derived hiPS-CMs had lower mitochondrial activity.

We next aimed to elucidate the mechanism of the decreased ΔΨm in the patient-derived hiPS-CMs. High-resolution respirometry analysis of digitonin-permeabilized cells demonstrated that each complex I- and complex I + II -dependent OCRs were significantly lower in patient-derived hiPS-CMs than in control (Fig. 2c, d). However, when the abundance of complexes I, III and IV was determined with reference to complex II by blue native polyacrylamide gel electrophoresis, the expression of each complex was maintained (Supplementary Fig. S4). Further measurement of the enzyme activity of each respiratory chain complex, as previously described,^50^ indicated no apparent decrease in the activity of patient-derived hiPS-CMs (Supplementary Table S2). To assess the availability of electron carriers in the mitochondrial respiratory chain, we quantified the coenzyme Q: ubiquinone (CoQ) levels using a liquid chromatograph-tandem mass spectrometer. Although the total CoQ levels did not decrease, the ratio of reduced ubiquinol (CoQH2) to total CoQ decreased in patient-derived hiPS-CMs (Supplementary Table S3). These results indicate that the decrease in OXPHOS capacity leads to possibly a lower mitochondrial activity in patient-derived hiPS-CMs.

### Patient-derived hiPS-CMs exhibit reduced ATP production

**Fig. 3:**
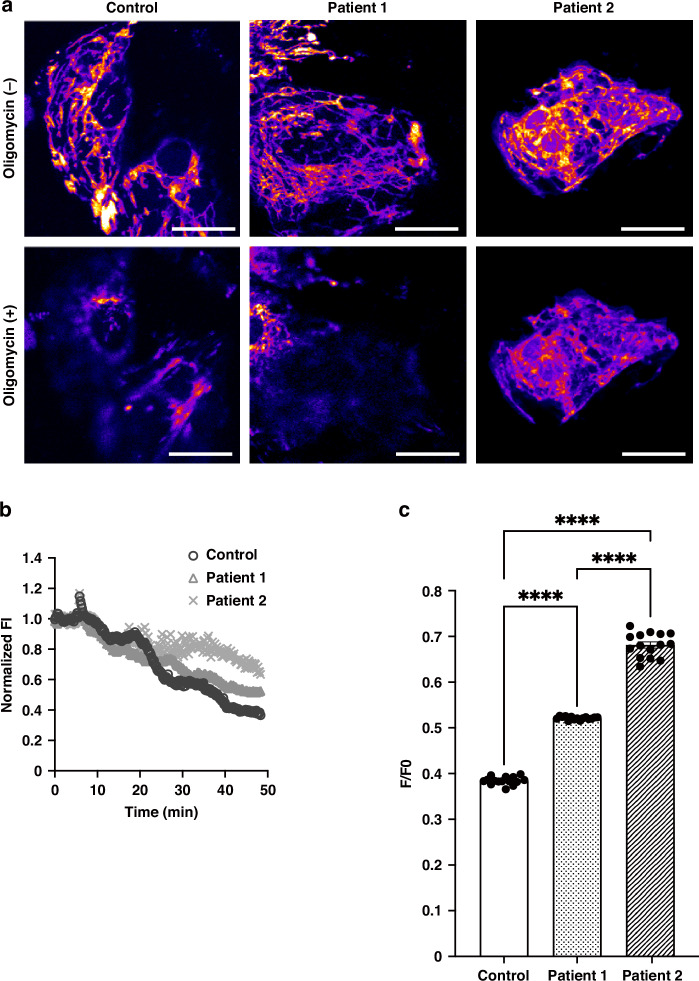
Evaluation of mitochondrial ATP production in patient-derived hiPS-CMs using MitoMAR. **a** Representative fluorescence images (LUT fire) of hiPS-CMs expressing MitoMAR before and 50-min after 25 μM oligomycin administration. Scale bar: 20 μm. **b** Time lapse experiments of each hiPS-CMs expressing MitoMAR with oligomycin treatment (circular dots: control, triangular dots: patient 1, cross dots: patient 2). Oligomycin was added at 0 min (F0). The averages of normalized fluorescence intensity (F/F0) per single cell (*n* = 15 cells) are plotted at each time point. **c** Average F/F0 of MitoMAR after oligomycin administration. The bar graph is obtained from a data set relevant to **b**. The normalized FI in the hiPS-CMs of patients 1 and 2 is significantly higher than that in control (control: 0.384 ± 0.002 [*n* = 15], patient 1: 0.521 ± 0.001 [*n* = 15], patient 2: 0.681 ± 0.007 [*n* = 15]). *n* = number of hiPS-CMs. ****p < 0.0001.

To evaluate mitochondrial ATP-producing capacity, we visualized mitochondrial ATP in live hiPS-CMs using an ATP indicator, mitochondrial targeting MaLionR (MitoMAR^43^). The hiPS-CMs were treated with 25 μM oligomycin (ATP synthetase inhibitor), and time lapse images of fluorescence decay were captured (Fig. 3a, b). The changes in fluorescence intensity before and 50 min after oligomycin administration were evaluated. In this assay, a higher F/F0 ratio represents a smaller ATP decrease, possibly indicating a lower capacity of ATP production, after oligomycin administration.^43^ As shown in Fig. 3c, both of the patient-derived iPS-CMs demonstrated higher F/F0 ratio, indicating less capacity of mitochondrial ATP production compared to control. These results were consistent with the results of OCR measurements.

### Patient-derived hiPS-CMs exhibit abnormal Ca^2+^ dynamics

**Fig. 4:**
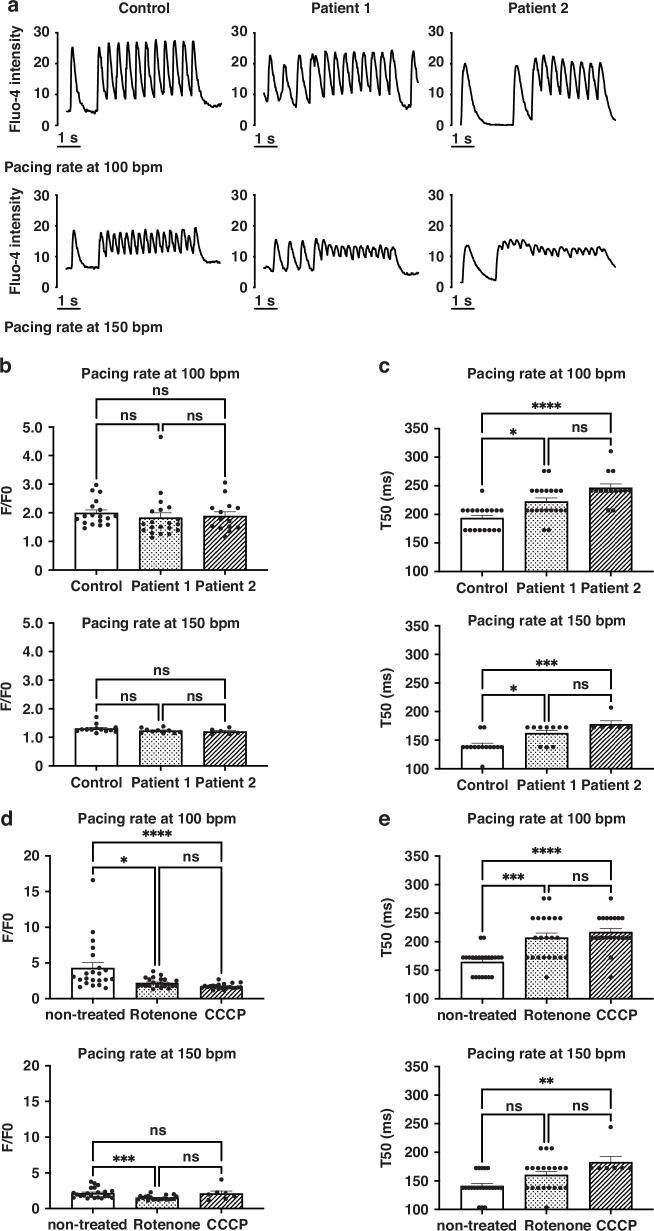
Evaluation of intracellular Ca^2+^ kinetics in patient-derived hiPS-CMs. **a** Representative fluorescence intensity waveforms of Fluo-4, an intracellular calcium indicator, in beat rate-controlled hiPS-CMs. Pacing rates are at 100 bpm (upper) and 150 bpm (lower), respectively. **b**, **c** The ratio of peak-to-base fluorescence intensity (F/F0) and 50% time to decay (T50) of Fluo-4 fluorescence intensity in beat rate-controlled hiPS-CMs. Pacing rates are at 100 bpm (upper) and 150 bpm (lower), respectively. There is no significant difference in F/F0 between control- and patient-derived hiPS-CMs shown in **b** (control: 1.996 ± 0.107 [*n* = 18], patient 1: 1.828 ± 0.175 [*n* = 20], patient 2: 1.886 ± 0.146 [*n* = 14] at pacing rate 100 bpm; control: 1.305 ± 0.040 [*n* = 13], patient 1: 1.228 ± 0.027 [*n* = 10], patient 2: 1.203 ± 0.039 [*n* = 6] at pacing rate 150 bpm). The T50 is significantly longer in patient-derived hiPS-CMs shown in **c** (control: 193.50 ± 4.94 [*n* = 18], patient 1: 222.40 ± 6.37 [*n* = 20], patient 2: 246.3 ± 7.10 [*n* = 14] ms at pacing rate 100 bpm; control: 140.40 ± 4.37 [*n* = 14], patient 1: 162.10 ± 5.27 [*n* = 10], patient 2: 178.10 ± 5.75 [*n* = 6] ms at pacing rate 150 bpm. **d**, **e** Effects of mitochondrial inhibitors on F/F0 and T50 in beat rate-controlled control hiPS-CMs. Pacing rates are at 100 bpm (upper) and 150 bpm (lower), respectively. Cells were pretreated with rotenone, a complex I inhibitor, for 1 h and with CCCP, an OXPHOS uncoupler, for 3 h. F/F0 shown in (**d**) (non-treated: 4.30 ± 0.74 [*n* = 22], rotenone: 2.22 ± 0.14 [*n* = 22], CCCP: 1.73 ± 0.08 [*n* = 21] ms at pacing rate 100 bpm; non-treated: 2.16 ± 0.15 [*n* = 22], rotenone: 1.51 ± 0.07 [*n* = 20], CCCP: 2.10 ± 0.36 [*n* = 7] ms at pacing rate 150 bpm). T50 shown in (**e**) (non-treated: 164.60 ± 4.50 [*n* = 22], rotenone: 206.90 ± 8.18 [*n* = 22], CCCP: 216.3 ± 6.36 [*n* = 21] ms at pacing rate 100 bpm; non-treated: 141.10 ± 4.49 [*n* = 22], rotenone: 160.30 ± 6.23 [*n* = 20], CCCP: 182.70 ± 10.25 [*n* = 7] ms at pacing rate 150 bpm). *n* = number of CMs. *p < 0.05, **p < 0.01, ***p < 0.001, ****p < 0.0001.

Intracellular Ca^2+^ kinetics was evaluated to assess cardiomyocyte function. The fluorescence intensity waveforms were recorded (Fig. 4a), and F/F0 (an index of calcium release from the endoplasmic reticulum) and T50 (an index of Ca^2+^ reuptake) assessed by beat rate-controlled hiPS-CMs. The F/F0 ratio in patient-derived hiPS-CMs was not different from that in control (Fig. 4b), whereas T50 was significantly prolonged (Fig. 4c). There was no obvious difference in SERCA2a mRNA expression between patient-derived and control hiPS-CMs (Supplementary Table S4).

### Reduced Ca^2+^ reuptake capacity is caused by mitochondrial ATP deficiency

To show that T50 prolongation was the result of mitochondrial ATP deficiency, we administered mitochondrial inhibitors to control hiPS-CMs to assess if the inhibitors caused T50 prolongation. Control hiPS-CMs were treated with 0.5 μM rotenone (a complex I inhibitor) for 1 h and with 2.5 μM carbonyl cyanide m-chlorophenylhydrazone (CCCP, an OXPHOS uncoupler) for 3 h. Then, we measured F/F0 and T50 of treated and non-treated control hiPS-CMs. F/F0 was significantly decreased and T50 was significantly longer in both the rotenone and CCCP groups than in the non-treated group (Fig. 4d, e).

### Patient-derived hiPS-CMs show reduced contractile and diastolic function

**Fig. 5:**
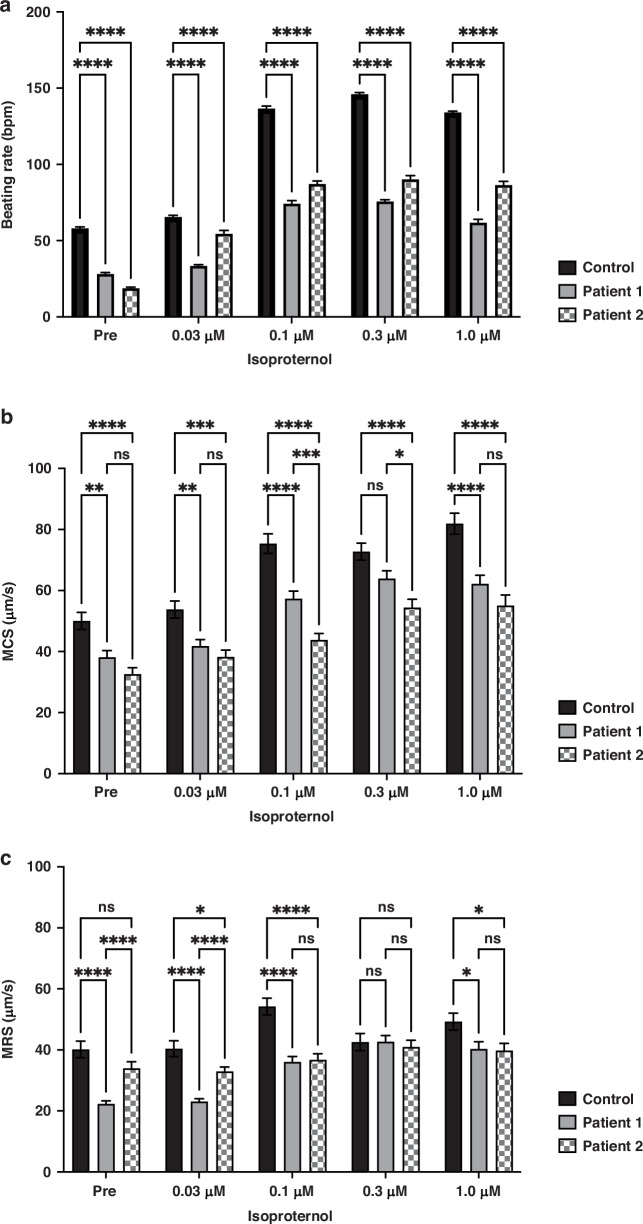
Evaluation of contractile and diastolic function of patient-derived hiPS-CMs. **a**–**c** The beating of hiPS-CMs was captured and the contractile and diastolic speeds were plotted (**a**). The increase in beating rate with isoproterenol titration is less in patient-derived hiPS-CMs than that in control. (beating rate Pre: 58.00 ± 1.02 vs. 28.15 ± 0.94 vs. 18.79 ± 0.71; 0.03 μM: 65.50 ± 1.03 vs. 33.45 ± 0.86 vs. 54.47 ± 2.24; 0.1 μM: 136.52 ± 1.72 vs. 74.23 ± 2.06 vs. 87.14 ± 2.00; 0.3 μM: 145.98 ± 1.05 vs. 75.74 ± 1.11 vs. 90.24 ± 2.44; 1.0 μM: 133.92 ± 0.98 vs. 61.75 ± 2.25 vs. 86.34 ± 2.51 bpm (control [*n* = 48] vs. patient 1 [*n* = 48] vs. patient 2 [*n* = 48])). *n* = number of fields of view. ****p < 0.0001. Changes in MCS and MRS with isoproterenol administration (**b**, **c**). Both MCS and MRS are lower in patient-derived hiPS-CMs than those in control (MCS Pre: 50.04 ± 2.81 vs. 38.10 ± 2.22 vs. 32.62 ± 2.10; 0.03 μM: 53.77 ± 2.78 vs. 41.79 ± 2.15 vs. 38.21 ± 2.28; 0.1 μM: 75.35 ± 3.21 vs. 57.32 ± 2.49 vs. 43.82 ± 2.13; 0.3 μM: 72.73 ± 2.79 vs. 63.88 ± 2.62 vs. 54.39 ± 2.78; 1.0 μM: 81.89 ± 3.44 vs. 62.20 ± 2.77 vs. 55.06 ± 3.47 μm/s (control [*n* = 48] vs. patient 1 [*n* = 48] vs. patient 2 [*n* = 48]), and MRS Pre: 40.14 ± 2.73 vs. 22.28 ± 1.04 vs. 33.96 ± 2.13; 0.03 μM: 40.40 ± 2.60 vs. 23.07 ± 0.92 vs. 32.92 ± 1.47; 0.1 μM: 54.21 ± 2.72 vs. 36.03 ± 1.81 vs. 36.74 ± 1.99; 0.3 μM: 42.55 ± 2.80 vs. 42.67 ± 2.05 vs. 40.92 ± 2.24; 1.0 μM: 49.27 ± 2.74 vs. 40.33 ± 2.32 vs. 39.76 ± 2.40 μm/s (control vs. patient 1 vs. patient 2)). *n* = number of fields of view. *p < 0.05, **p < 0.01, ***p < 0.001, ****p < 0.0001.

Cardiomyocyte motion, waveforms of contraction and relaxation speeds were captured using a high-precision live imaging system (Supplementary Movie S1, Supplementary Fig. S5). We measured MCS as the contractile index and MRS as the diastolic index under steady state. In the clinical course, cardiac dysfunction in both patients had occurred at the onset of the infectious disease. We hypothesized that mitochondrial dysfunction in patient-derived hiPS-CMs would be more apparent under conditions of increased oxygen demand, such as fever and tachycardia caused by infectious diseases. We, therefore, decided to induce tachycardia with isoproterenol and evaluate changes in systolic and diastolic function. To induce tachycardia, isoproterenol was administered, and the concentration was gradually increased (0.03, 0.1, 0.3, and 1.0 μM). Although there was an increase in beat rate with isoproterenol administration in both patient-derived and control hiPS-CMs, the beat rate increase was significantly lower in patient-derived hiPS-CMs than in control (Fig. 5a). In patient-derived hiPS-CMs, there were significant decreases in MCS and MRS not only at steady state, but also under isoproterenol administration (Fig. 5b, c).

To find out the difference in electrical activity, the extracellular electrical potential was measured using a multi-electrode array system. No obvious abnormalities in the extracellular potential waveforms or significant differences in the corrected field potential duration were noted between control and patient-derived hiPS-CMs (Supplementary Fig. S6a, b).

## Discussion

In this study, we established hiPSCs from two pediatric patients with *DNM1L* mutation who developed cardiac dysfunction and differentiated them into hiPS-CMs. Mitochondrial morphology and function and cardiomyocyte function were then analyzed in patient-derived hiPS-CMs. The results showed that the mitochondria in patient-derived hiPS-CMs exhibited a significantly elongated morphology, unlike the control. These morphological abnormalities are consistent with those reported in fibroblasts with other *DNM1L* mutations.^51^ Furthermore, overexpression of wild-type Drp1 in patient-derived hiPS-CMs changed elongated mitochondrial morphology to a short, fragmented form. These results suggest that the missense mutations in both patients cause abnormal mitochondrial morphology due to a dominant-negative effect, similar to the previously reported missense mutations.^11,14,15,17,18,24,27^

No increased expression of Drp1 was observed in patient 1-derived hiPS-CM, whereas its expression was elevated in patient 2-derived hiPS-CM. Mitochondrial elongation and dysfunction were more pronounced in patient 2-derived hiPS-CM than in patient 1-derived hiPS-CM, suggesting a compensatory increase in Drp1 expression. Dephosphorylation of Drp1 at S616 and phosphorylation at S637 are predicted to suppress mitochondrial fission.^48,49^ However, there was no significant difference in phosphorylation at S616 between control and patient-derived hiPS-CMs. Furthermore, phosphorylation at S637 was significantly lower in patient 2-derived hiPS-CM. These results indicate that the *DNM1L* mutations in patients 1 and 2 suppresses mitochondrial fission by a mechanism distinct from the phosphorylation regulation of Drp1. The effect of *DNM1L* mutations on proteins involved in mitochondria dynamics will be investigated in our future studies.

We next assessed mitochondrial function in patient-derived hiPS-CMs. JC-1 staining revealed a decrease in the ΔΨm, indicating poor mitochondrial activity. Moreover, OCR and ATP production were decreased in patient-derived hiPS-CMs although respiratory chain enzyme expression and activity were not significantly reduced compared to control.

Based on these results, CoQ, responsible for the transfer of electrons from complexes I and II to complex III,^52^ was analyzed. The CoQH2/CoQ ratio was decreased in patient-derived hiPS-CMs, even though the amount of total CoQ was not different from that in control, indicating the possibility that the reduction in CoQH2 level inhibited electron transfer, leading to decreased ATP production. The mechanism by which CoQH2/CoQ was decreased by *DNM1L* mutations needs to be clarified.

Previous studies have shown that suppression of Drp1 inhibits mitophagy.^53,54^ It is speculated that inhibition of mitophagy leads to the accumulation of elongated and damaged mitochondria by impairing mitochondrial clearance, resulting in reduced membrane potential and decreased ATP production. Impairment of mitophagy in patient-derived hiPS-CM needs to be investigated.

Cardiomyocyte dysfunction was assessed by analyzing intracellular Ca^2+^ dynamics and contractile and diastolic funtion. In the analysis of Ca^2+^ dynamics, T50 was significantly prolonged in patient-derived hiPS-CMs. There was no significant difference in the mRNA levels of *SERCA2a*, which is involved in Ca^2+^ reuptake in the sarcoplasmic reticulum, suggesting a functional reduction of SERCA2a in patient-derived hiPS-CMs. Further analysis of protein levels is needed to assess the impact of quantitative variation in SERCA2a. Previous studies have revealed that reduced expression or deregulation of SERCA2a leads to myocardial dysfunction.^55–58^ As SERCA2a is ATP dependent^59^ and consumes large amounts of ATP,^60^ we speculate that insufficient ATP in *DNM1L*-mutated cardiomyocytes leads to SERCA2a dysfunction, resulting in cardiac dysfunction. To demonstrate that decreased mitochondrial ATP production leads to a loss of SERCA2a function, we examined whether the addition of mitochondrial inhibitors to control-derived hiPS-CMs altered T50. We found that T50 was prolonged after treatment with either rotenone, a complex I inhibitor, or CCCP, a de-conjugating agent that lowered the ΔΨm. These results support the hypothesis that T50 prolongation in patient-derived hiPS-CMs is possibly due to the dysfunction of SERCA2a secondary to ATP deficiency. However, function of SERCA2a is modified with various protein modifications such as phosphorylation, SUMOylation, and oxidative modification or protein-protein interaction such as SERCA2a-phospholamban and SERCA2a-dwarf open reading frame.^61^ The cause of SERCA2a dysfunction in patient-derived hiPS-CMs could not be explained by mitochondrial dysfunction alone. The effects of *DNM1L* mutations on these regulatory systems should also be verified. In addition, the F/F0 was decreased following mitochondrial inhibitor administration. We hypothesized that Ca^2+^ reuptake by SERCA2a is rapidly lowered by ATP inhibitors, resulting in a rapid decrease in the Ca^2+^ concentration in the sarcoplasmic reticulum. Consequently, the probability of ryanodine receptor opening driven by the Ca^2+^ gradient is reduced.

We further quantified the contractile and diastolic function using a high-performance imaging system. Contractile and diastolic function were significantly lower in patient-derived hiPS-CMs than in control at steady state. The contractility of patient-derived hiPS-CMs was lower than that of control cells even before stimulation with isoproterenol. These results are consistent with previous reports,^62^ as that modulation of SERCA2 capacity of Ca^2+^ transport alters Ca^2+^ levels in unstimulated condition and causes cardiac dysfunction. Furthermore, under isoproterenol administration, patient-derived hiPS-CMs showed a smaller response to isoproterenol as increases in the beat rate and contractile and diastolic performance.

There were no obvious extracellular electrical potential abnormalities in patient-derived hiPS-CMs. These results indicate that although cardiomyocyte oxygen demand increases with isoproterenol administration, insufficient ATP production does not meet the oxygen demand in patient-derived hiPS-CMs, resulting in a small response to isoproterenol. The reduced contractile and diastolic function were likely due to reduced mitochondrial ATP production and abnormal Ca^2+^ handling.

As described in the previous reports, although many pediatric cases with neurological impairments have been reported, there are only a few reports on cardiac dysfunction caused by *DNM1L* mutations. The human central nervous system consists of nearly 100 billion neurons^63^ and is a highly oxygen-consuming organ. However, the density of somatic mitochondria in hippocampal neurons is approximately less than 10%.^64^ We consider that the low mitochondrial density in neurons makes them susceptible to mitochondrial quality loss owing to *DNM1L* mutations, resulting in early neurological damage. Meanwhile, cardiomyocytes have a high mitochondrial density of ~50% per cell,^65^ which means that ATP production can be compensated by the number of mitochondria. Thus, it can be assumed that functional impairments owing to insufficient ATP production is not significant in cardiomyocytes under normal condition. In both the patients in this study, cardiac dysfunction was probably worsened by infection. We speculate that cardiac dysfunction is more likely to occur when ATP production is insufficient to meet the increased oxygen demand, such as during fever and tachycardia.

With regard to cardiac dysfunction caused by *DNM1L* mutations, the correlation between genotype and phenotypic severity remains unclear. In this study, patient 1 had a de novo point mutation in the stalk domain, while patient 2 had a de novo point mutation in the variable domain. With respect to the clinical course, patient 2 had a more severe cardiac dysfunction. Some results of this study were consistent with the clinical course, with more pronounced mitochondrial elongation (Fig. 1c), decreased ATP production (Fig. 3c), and decreased contractile function under isoproterenol titration (Fig. 5b) being observed in hiPS-CMs derived from patient 2 compared to those from patient 1. Gawlowski et al. reported that O-linked N-acetyl-glucosamine glycosylation (O-GlcNAcylation) regulated Drp1 function in cardiomyocytes.^66^ They found that Drp1 was O-GlcNAcylated at threonine 585 (T585) and threonine 586 (T586) and increased O-GlcNAcylation attenuated the phosphorylation of S637 in Drp1, resulting in increased levels of the GTP-bound active form of Drp1, which promoted mitochondrial fragmentation. A missense mutation (Thr586Lys) in *DNM1L* gene in patient 2 was expected to inhibit O-GlcNAcylation and enhance downstream S637 phosphorylation, but our experimental results showed no enhancement in S637 phosphorylation. A mechanism for suppression of Drp1 function other than regulation of S637 phosphorylation with O-GlcNAcylation needs to be elucidated.

The limitation of this study is the small number of included patients, which may limit the generalizability of the study results. In addition, the use of control cells with different genetic backgrounds may induce unpredictability and confounding effects. Future work will require (1) collecting samples with other *DNM1L* mutations to investigate the correlation between the site of mutation and the severity of the disease and (2) using gene editing tools such as CRISPR-Cas9 to generate homogeneous control cell lines with similar genetic backgrounds.

In conclusion, mitochondrial fission by Drp1 is an important for the process in the removal of aging mitochondria and for the maintenance of mitochondrial quality to keep ATP levels in cardiomyocytes in patients with *DNM1L* mutation. Insufficient ATP production leads to SERCA2a dysfunction, resulting in reduced contractile and diastolic function of cardiomyocytes. Controlling this process would be a target of novel therapies for the patients.

## Supplementary information

Supplementary Movie S1

Supplementary Movie S2

Supplementary Movie S3

Supplementary Movie S4

Supplementary Movie S5

Supplementary Movie S6

Supplementary Movie S7

Supplementary Movie S8

Supplementary Movie S9

Supplementary Movie S10

Supplementary Movie S11

Supplementary Movie S12

Supplementary Movie S13

Supplementary Movie S14

Supplementary Movie S15

Supplementary information

## Data Availability

The datasets generated during and/or analyzed during the current study are available from the corresponding author on reasonable request.
