# Supplementary material for: Cardiac dysfunction due to mitochondrial impairment assessed by human iPS cells caused by DNM1L mutations
Source: Pediatr Res. 2025 Apr 23;98(5):1929–40. doi: 10.1038/s41390-025-04045-6 (PMC12602321; doi:10.1038/s41390-025-04045-6)
Supplement: Supplementary file 16 — Supplementary information [file 41390_2025_4045_MOESM16_ESM.pdf]

Supplementary Materials

Title: Cardiac dysfunction due to mitochondrial impairment assessed by human iPS cells caused by *DNM1L* mutations

Supplementary Methods

Immunocytochemistry and FACS analysis of hiPS-CMs

Immunostaining of cardiac troponin T

hiPS-CMs were reseeded in a 35-mm glass-bottom dish and fixed in phosphate-buffered saline (PBS) containing 4% paraformaldehyde and treated with PBS containing 0.1% Triton X-100 (NACALAI TESQUE, Kyoto, Japan). The cells were then stained with a primary antibody (cTnT; 1:200, Thermo Fisher Scientific, Waltham, MA) and incubated overnight at 4°C. Next, hiPS-CMs were stained with a secondary antibody (Alexa Fluor 488-conjugated donkey anti-goat IgG, 1:2000, Thermo Fisher Scientific) and incubated at 25°C for 1 h. The nuclei were stained with Nucblue™ Fixed Cell Reagent™ (Thermo Fisher Scientific). Fluorescence microscopy images were obtained using a confocal microscope (Leica TCS SP8X) at 40× magnification (Fig. S7a).

FACS analysis of cardiac troponin T

hiPS-CMs were fixed in BD Cytofix/Cytoperm (BD Biosciences, San Jose, CA) for 20 min and washed with BD Perm/Wash, stained with an anti-cTnT antibody (1:100; Thermo Fisher Scientific), incubated overnight at 4°C, and labelled with a secondary antibody (Alexa Fluor 488-conjugated donkey anti-goat IgG,1:2000, Thermo Fisher Scientific). Data were acquired using a FACSVerse flow cytometer (BD Biosciences) and analyzed using FACSuite software (BD Biosciences). We obtained differentiated cardiomyocytes with a purity of approximately >80% (Fig. S7b).

Western blot analysis of Drp1

Cells were lysed with RIPA buffer (50 mM Tris-HCl [pH 7.5], 150 mM NaCl, 1% Triton X-100, 0.5% sodium deoxycholate, 0.1% SDS, 5 mM EDTA) containing protease inhibitor (Sigma-Aldrich) and phosphatase inhibitor cocktails (Sigma-Aldrich). Equal amounts of protein were separated by SDS-polyacrylamide gel electrophoresis. Proteins were transferred to a polyvinylidene difluoride membrane (GE Healthcare, Chicago, IL) and stained with Ponceau S (Thermo Fisher Scientific). After capturing images, the membrane was destained and blocked with 5% skim milk in Tris-HCl buffered saline and incubated with a primary antibody overnight at 4°C. Primary antibodies against pDrp1 at Ser 616 and pDrp1 at Ser 637 were purchased from Cell Signaling Technology (Danvers, MA) and anti-Drp1 antibody was from abcam (Cambridge, UK). After washing, the membrane was incubated with

a horseradish peroxidase-conjugated secondary antibody (Cell Signaling Technology) for 1 h at room temperature and treated with a chemiluminescence detection regent (ImmunoStar, FUJIFILM WAKO). Positive signals were visualized using a chemiluminescence imaging system (FUSION; Vilber Lourmat, Paris, France).

BN-PAGE analysis

BN-PAGE analysis was performed as previously reported with a slight modification.[1] Briefly, hiPS-CMs pellets were suspended in mitochondria isolation buffer A (20 mM HEPES, 70 mM sucrose, 220 mM mannitol, 1 mM EDTA, 2 mg/mL bovine serum albumin, 1× protease inhibitor, pH 7.4) and homogenized on ice. The homogenates were then centrifuged at 700 *g* at 4°C for 5 min to separate the nuclei and cytoplasm. The supernatant was further centrifuged at 1000 *g* at 4°C for 10 min to precipitate the mitochondrial pellet. The mitochondrial pellets were washed twice with buffer B (220 mM mannitol, 20 mM HEPES, 70 mM sucrose, 1 mM EDTA, pH 7.4, 1× protease inhibitor cocktail) to remove debris. Mitochondrial samples were dissolved in NativePAGE sample buffer containing 0.5% Triton-X and loaded on electrophoresed on 3–14% acrylamide gel. The samples were electrophoresed using 1× Native PAGE Running buffer system (Invitrogen). For immunoblotting analyses, anti-NDUFA9 (Complex I) (Invitrogen), anti-SDHA (Complex II) (Invitrogen), anti-UQCRC1 (Complex III) (Invitrogen), and anti-MTCO1 (Complex IV) (Invitrogen) monoclonal antibodies were used as primary antibodies. An anti-mouse IgG peroxidase antibody (Sigma Aldrich) was used as the secondary antibody. Complex II has been used as an internal quality marker.[2]

Quantification of CoQ and CoQH2

After incubation, hiPS-CMs were washed twice in PBS, pelleted, resuspended at 3×10^6 in 700 µL 2-propanol, frozen in acetone on dry ice, and stored on dry ice. For the analysis, 300 µL of 2-propanol containing an internal standard of ubiquinone-7 at 50 ng/mL was added to 200 µL of the cell suspension, stirred with a vortex mixer for 2 min, and centrifuged for 5 min at 12,000 rpm. The supernatant was diluted 5-fold in 2-propanol:methanol (4:5, v/v), and 10 µL of the diluted solution was injected into a liquid chromatograph-tandem mass spectrometer (LC/MS/MS) system.

CoQ and CoQH2 levels in the cells were determined using an LC/MS/MS method described by Ruiz Jimenez et al[3] with minor modifications. Briefly, detection and quantification were performed using a Triple Quad 5500 LC-MS/MS System (AB SCIEX,

Framingham, MA) equipped with a Turbo Ion Spray electrospray ionization source and a Nexera UFLC system (Shimadzu, Kyoto, Japan). Chromatographic separation was performed on a YMC-UltraHT Pro C18 column, 50 mm × 2.0 mm I.D., 2.0 μm particle size (YMC, Kyoto, Japan) maintained at 30°C. The mobile phase was methanol containing 5 mM ammonium formate:2-propanol-ultrapure water (50:47:3, v/v/v) pumped at a rate of 0.5 mL/min. The run time was 5 min per injection. Calibration curves were derived from the peak area ratios (analyte/internal standard) using weighted linear least-squares regression of the peak area ratio versus the concentration of the standards.

Quantitative real-time RT-PCR

RNA was extracted from hiPS-CMs using the RNeasy® Mini Kit (QIAGEN, Hilden, Germany). cDNA was synthesized using ReverTra Ace® qPCR RT Master Mix (TOYOBO, Osaka, Japan) according to the manufacturer's protocol. Quantitative mRNA expression was assessed using real-time polymerase chain reaction with TB Green® Premix Ex Taq ® II (TaKaRa Bio, Shiga, Japan). The samples were run in duplicate using ViiA™ 7 (Applied Biosystems, Waltham, MA). The oligonucleotide primer pairs are listed in Supplementary Table S5. The relative mRNA expression was normalized to Rps18.

Measurement of extracellular electrical potential of hiPS-CMs

hiPS-CMs were dissociated using 1 mg/dL collagenase B (Roche, Roswell) and Accumax (Innova Cell Technologies). The cell suspension (5 μL of approximately 5.0×10^4 cells) was placed onto a Matrigel-coated 24-well electrode plate and incubated at 37°C, 5% CO_2, for at least 7 days. The samples were maintained in iCell Cardiomyocyte Maintenance Medium (FUJIFILM Cellular Dynamics Inc., Madison, WI). We recorded the extracellular potential waveforms of hiPS-CMs using a Maestro Edge MEA system (AXION BIOSYSTEMS, Atlanta, GA). Field potential duration (FPD) was measured and corrected using the beating rate as previously described.[4] Corrected field potential duration (FPDc) was compared among the cell lines.

Supplementary References

1. Kohda, M., *et al.* A Comprehensive Genomic Analysis Reveals the Genetic Landscape of Mitochondrial Respiratory Chain Complex Deficiencies. *PLoS Genet* **12**, e1005679 (2016).
2. Ahmed, S.T., *et al.* Using a quantitative quadruple immunofluorescent assay to diagnose isolated mitochondrial Complex I deficiency. *Sci Rep* **7**, 15676 (2017).
3. Ruiz-Jiménez, J., Priego-Capote, F., Mata-Granados, J.M., Quesada, J.M. & Luque de Castro, M.D. Determination of the ubiquinol-10 and ubiquinone-10 (coenzyme Q10) in human serum by liquid chromatography tandem mass spectrometry to evaluate the oxidative stress. *J Chromatogr A* **1175**, 242-248 (2007).
4. Millard, D., *et al.* Cross-Site Reliability of Human Induced Pluripotent stem cell-derived Cardiomyocyte Based Safety Assays Using Microelectrode Arrays: Results from a Blinded CiPA Pilot Study. *Toxicol Sci* **164**, 550-562 (2018).

Supplementary Table S1: Summary of clinical findings for each patient

	Patient 1	Patient 2
Age of onset	6 month-old	20 month-old
Sex	Male	Male
Initial symptoms	growth retardation, hypotonia, and infantile spasm	fever, acute respiratory, and heart failure
Laboratory data	mildly elevated lactate/pyruvate ratio in the cerebrospinal fluid	metabolic acidosis with hyperlactemia, lactic acidosis, and hyperammonemia
Echocardiography	EF 56%<br>thickening of the myocardial wall and pericardial effusion at the onset of pneumonia the onset of pneumonia	EF 40%<br>decreased contractility
Outcome	death due to aspiration pneumonia	survival with neurological sequelae
Autopsy	fibrosis and degeneration of cardiomyocytes	not performed
Mutation site	c.1217T>C, p.Leu406Ser	c.1757C>A, p.Thr586Lys

Supplementary Table S2: Respiratory chain enzyme activity in patient-derived hiPS-CMs

	Complex I	Complex II	Complex II+III	Complex III	Complex IV	CS
Patient 1						
Crude activity (%)	98.8*	144.6	242.7	74.0	142.1	100.5
CS ratio (%)	98.4	143.9	241.6	73.7	141.4	-
Complex II ratio (%)	68.3	-	167.8	51.2	98.3	-
Patient 2						
Crude activity (%)	126.3	148.1	230.0	68.5	152.6	81.0
CS ratio (%)	156.0	182.9	284.0	84.5	188.4	-
Complex II ratio (%)	85.3	-	155.3	46.2	103.0	-

*Enzyme activities of each complex are presented as the percentage of control mean relative to citrate synthase (CS) activity.

Supplementary Table S3: Amount of CoQ10 in hiPS-CMs

	Control	Patient 1	Patient 2
Total CoQ (ng/mL/cell)*	1.21×10^{-4}	1.05×10^{-4}	1.58×10^{-4}
Ratio: CoQH2/ Total CoQ	0.72	0.17	0.42

*The values above are raw data. Number of cardiomyocytes, 3.1×10^6 in control, 5.6×10^6 in patient 1 and 5.0×10^6 in patient 2 were used.

Supplementary Table S4: mRNA expressions of SERCA2a receptor in hiPS-CMs

	Control	Patient 1	Patient 2	P-value
SERCA2a/Rps18	0.50 ± 0.18	0.35 ± 0.09	0.67 ± 0.11	0.35

Number of each cardiomyocyte sample: control n=4, patient 1 n=3, and patient 2 n=4. Rps18, ribosomal protein S18.

Supplementary Table S5: Primer pairs used for quantitative reverse transcription polymerase chain reaction (RT-PCR) of SERCA2a

SERCA2a (forward)	5′-TTGTGGCCCGAAACTACCTG-3′
SERCA2a (reverse)	5′-CTAACAACGCACATGCACGC-3′

Supplemental Figures

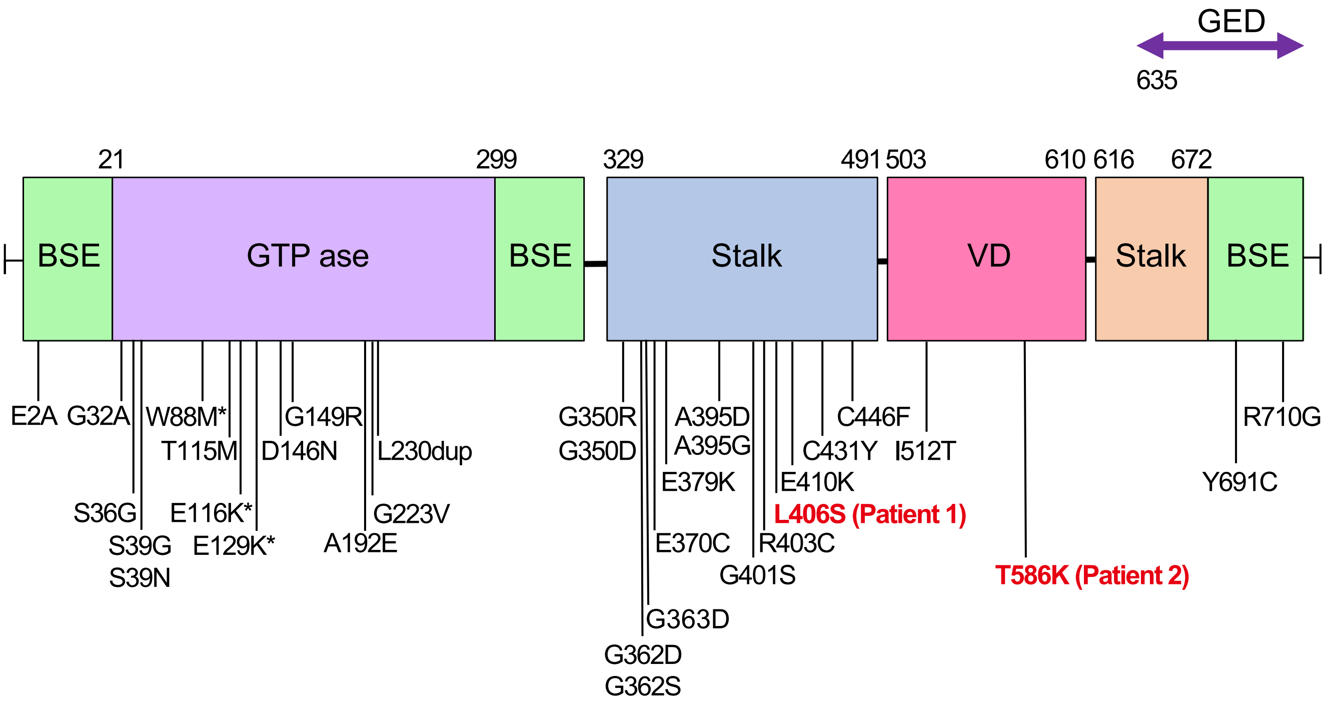

Fig. S1 Schematic representation of known *DNM1L* mutation sites and the domain organization. The mutation sites in patients 1 and 2 are shown in red. Organization: bundle signaling element (BSE), GTPase domain, stalk domain, variable domain (VD), and GTPase effector domain (GED).

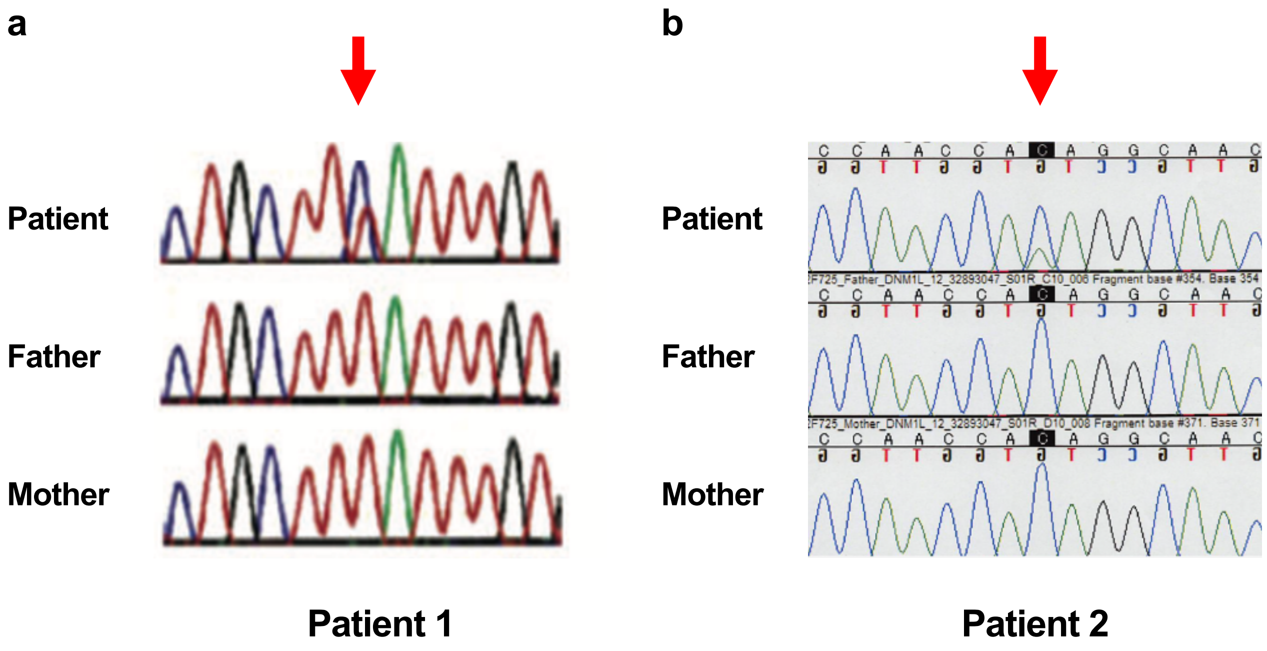

Fig. S2 ***DNM1L*** **mutation in each patient.**

a De novo missense mutation (c.1217T>C, p.Leu406Ser) in patient 1.

b De novo DNM1L mutation (c.1757C>A, p.Thr586Lys) in patient 2.

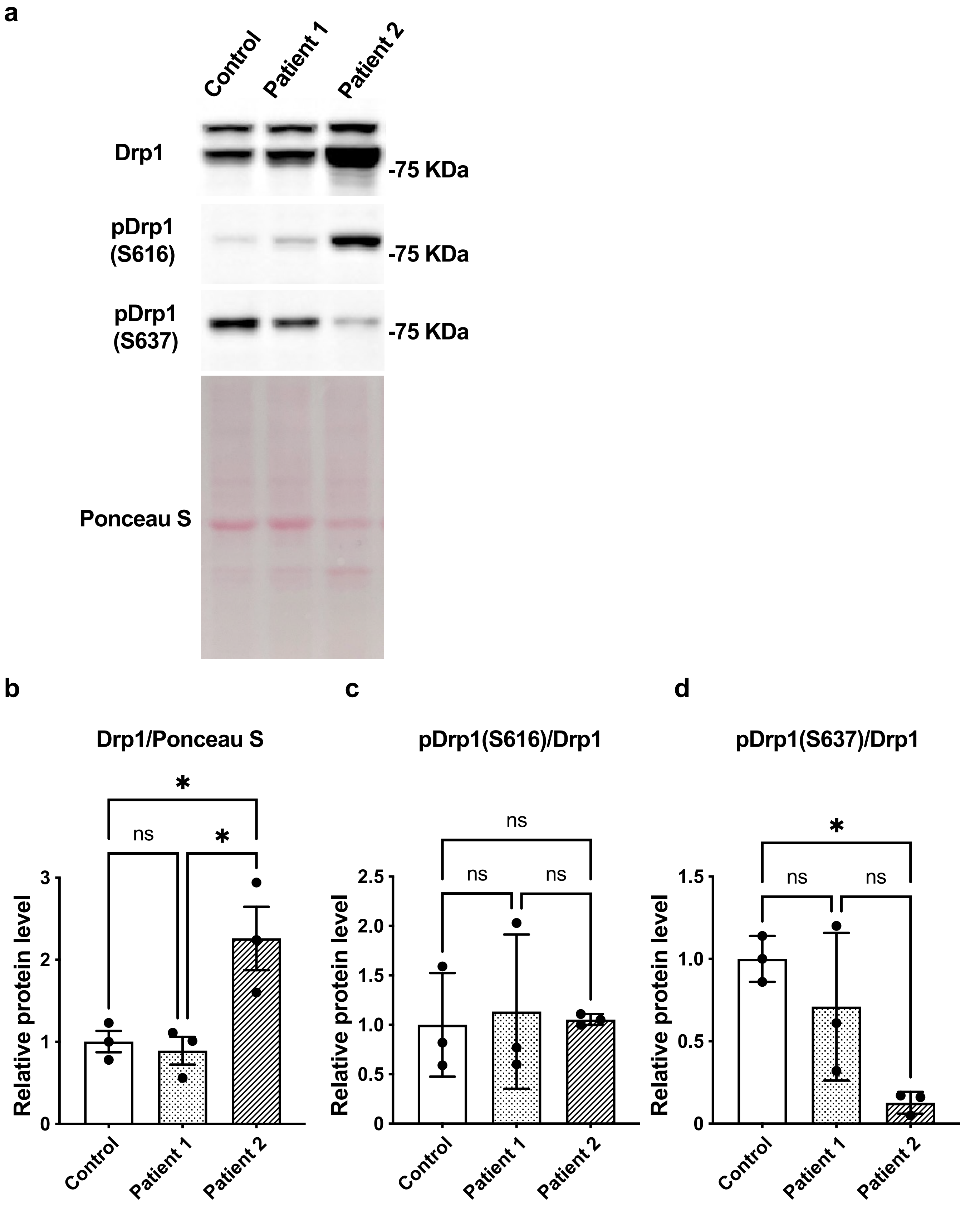

Fig. S3: Western blot analysis of Drp1 and pDrp1 in hiPS-CMs.

a Protein expression of Drp1 and pDrp1 was analyzed by western blotting. **b** Drp1/Ponceau S protein ratio was significantly lower in patient 2-derived hiPS-CMs compared to control (control: 1.00 ± 0.13 [n = 3], patient 1: 0.89 ± 0.17 [n = 3], patient 2: 2.26 ± 0.39 [n = 3]) n = number of cell pellets. *p < 0.05. **c, d** The ratio of pDrp1 S616/Drp1 was not significantly different from control in both patient-derived hiPS-CMs, while the pDrp1 S637/Drp1 ratio was significantly decreased in patient 2-derived hiPS-CMs. (pDrp1 S616/Drp1: control: 1.00 ± 0.30 [n = 3], patient 1: 1.13 ± 0.45 [n = 3], patient 2: 1.05 ± 0.03 [n = 3]) (pDrp1 S637/Drp1: control: 1.00 ± 0.08 [n = 3], patient 1: 0.71 ± 0.26 [n = 3], patient 2: 0.13 ± 0.04 [n = 3]). n = number of cell pellets. *p < 0.05.

a

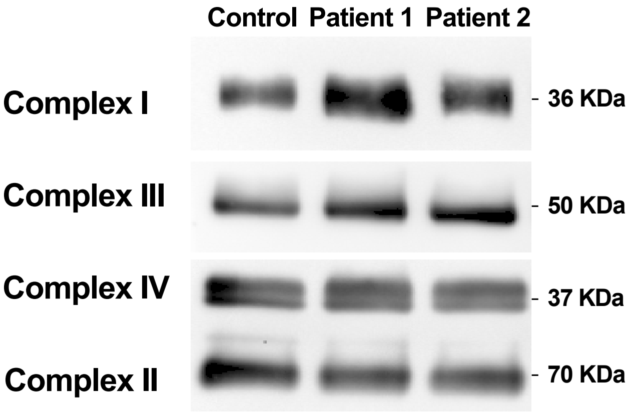

b

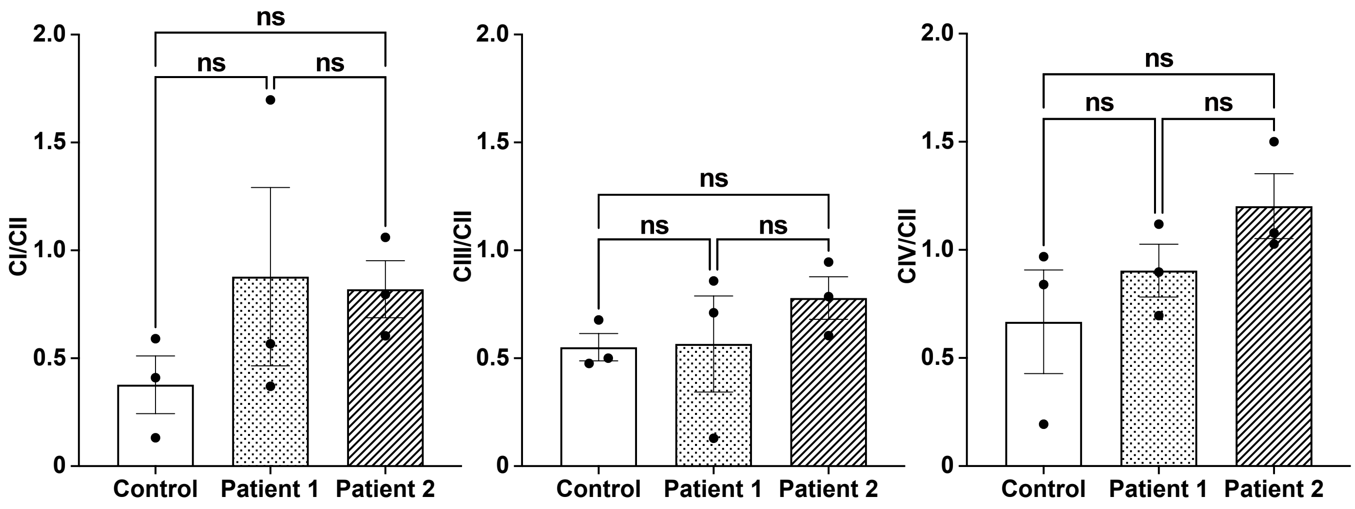

Fig. S4. Blue native polyacrylamide gel electrophoresis (BN-PAGE) analysis of hiPS-CMs.

a BN-PAGE of hiPS-CMs. **b** The ratio of the signal intensity of each complex (C) to the complex II is shown in the graph. (CI/CII: control: 0.38 ± 0.13 [n = 3], patient 1: 0.88 ± 0.41 [n = 3], patient 2: 0.82 ± 0.13 [n = 3]) (CIII/CI: control: 0.55 ± 0.06 [n = 3], patient 1: 0.57 ± 0.22 [n = 3], patient 2: 0.78 ± 0.10 [n = 3]) (CIV/CII: control: 0.67 ± 0.24 [n = 3], patient 1: 0.90 ± 0.12 [n = 3], patient 2: 1.20 ± 0.15 [n = 3]). n = number of cell pellets.

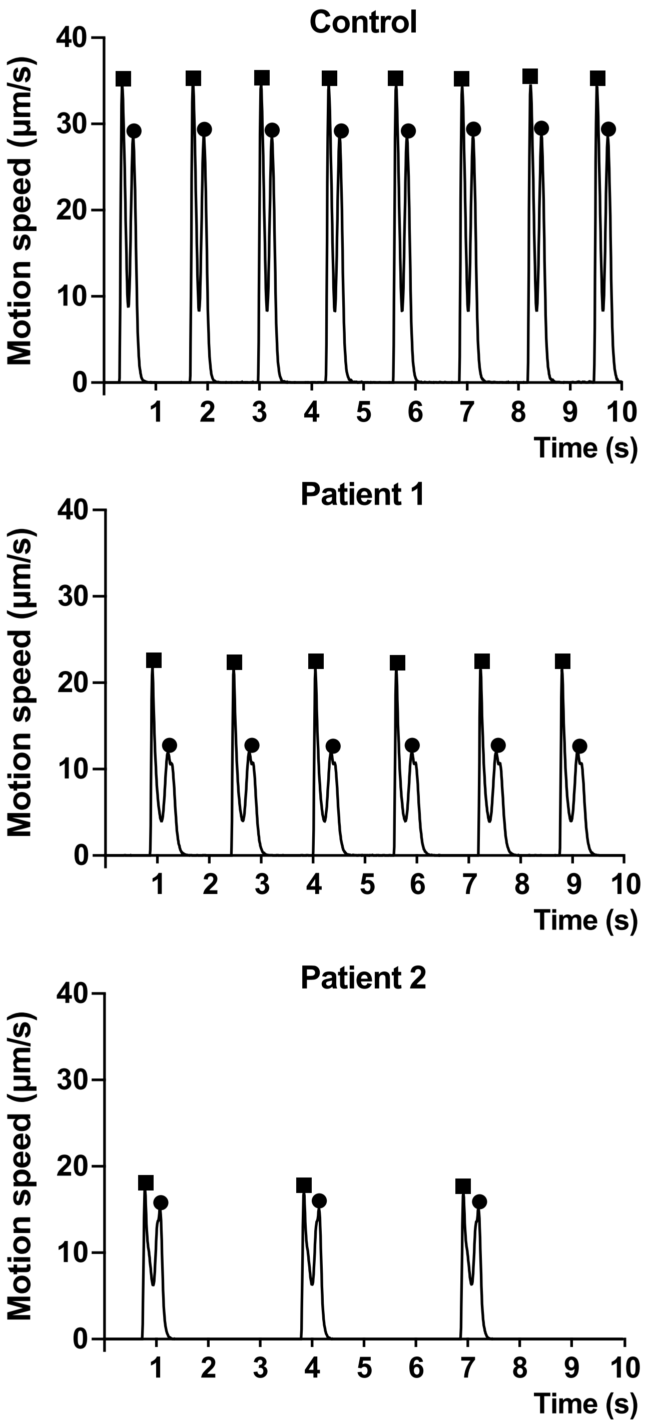

Fig. S5 Representative motion waveforms of hiPS-CMs at steady state.

Square dots indicate maximum contraction speed (MCS), and circular dots indicate maximum relaxation speed (MRS) (μm/s).

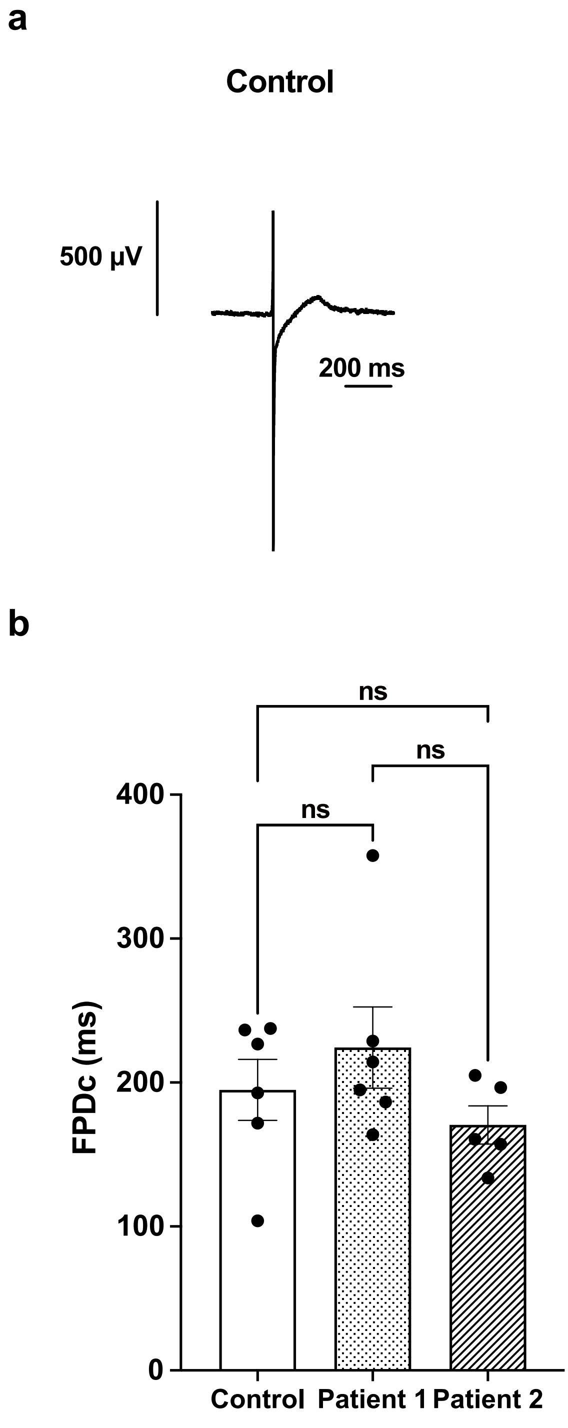

Fig. S6 Recording of extracellular electrical potential of hiPS-CMs using a multi-electrode array (MEA) system.

a Representative extracellular potential waveforms of hiPS-CMs. **b** No significant differences are found in corrected field potential duration (FPDc) at steady state. (control: 194.9 ± 21.1 [n = 6], patient 1: 224.3 ± 28.2 [n = 6], patient 2: 170.5±13.2 [n = 5] ms). n = number of electrode wells.

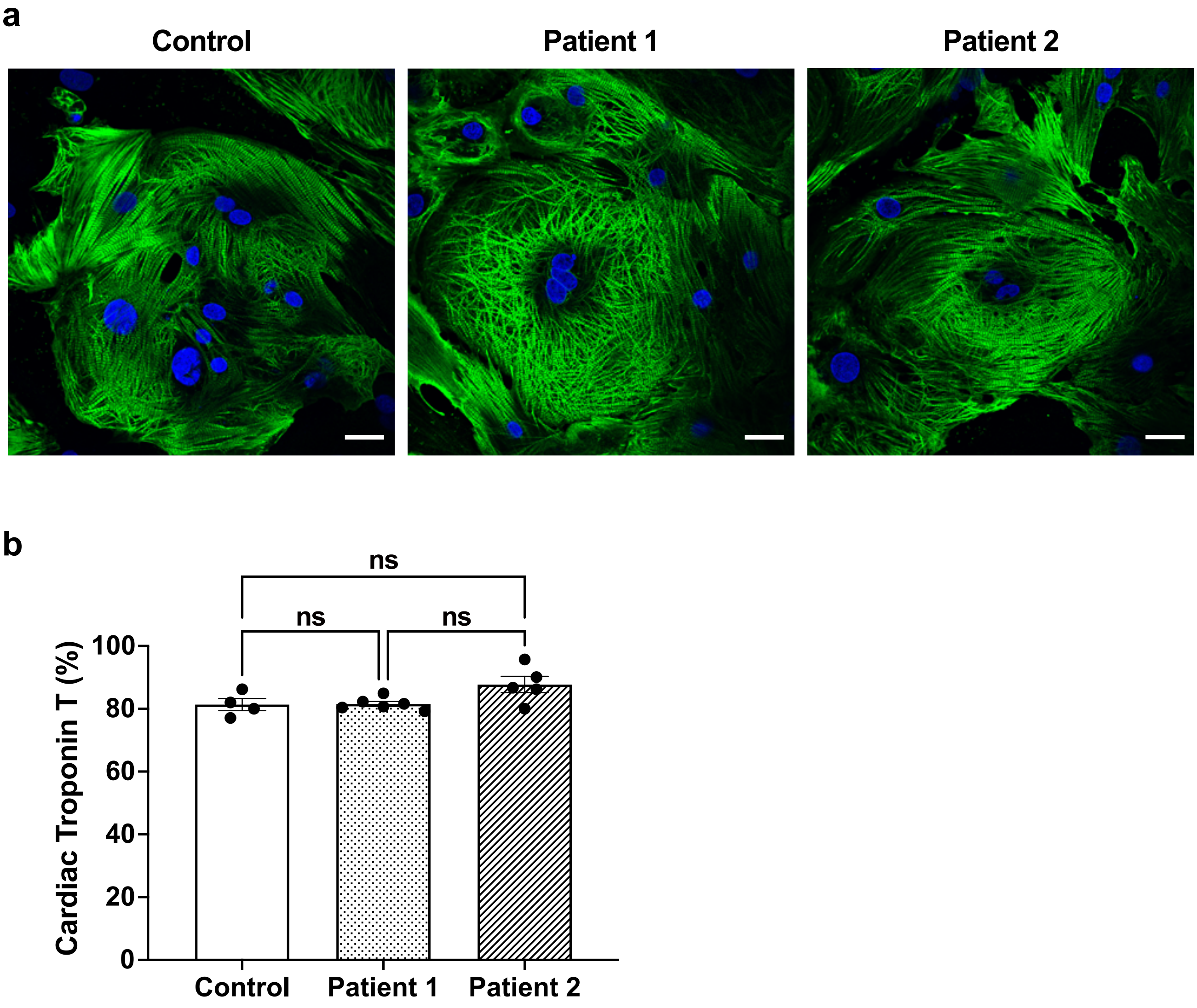

Fig. S7 Cardiac troponin T staining and FACS analysis of hiPS-CMs.

a Immunostaining of cardiac troponin T in control, Patient 1, and Patient 2-derived hiPS-CMs. Scale bar: 25 μm. **b** The average percentage of cardiac troponin T-positivity in FACS analysis.

The average positive rate was approximately >80%, with no significant difference among control, patient 1, and patient 2. (control: 81.34 ± 1.92 [n = 4], patient 1: 81.52 ± 0.80 [n = 6], patient 2: 87.74 ± 2.57 [n = 5] %). n = number of cell pellets.

Supplementary Legends for Video Files

Supplementary Movie S1. Representative movies of beating hiPS-CMs at steady state and after isoproterenol administration (Pre, 0.03, 0.1, 0.3, and 1.0 µM) captured by SI8000. Patient-derived hiPS-CMs showed lower contractile and diastolic function than control cardiomyocytes.
